# Transfoveal Micropulse Laser Treatment of Central Serous Chorioretinopathy within Six Months of Disease Onset

**DOI:** 10.3390/jcm8091398

**Published:** 2019-09-06

**Authors:** Maciej Gawęcki, Agnieszka Jaszczuk-Maciejewska, Anna Jurska-Jaśko, Małgorzata Kneba, Andrzej Grzybowski

**Affiliations:** 1 Dobry Wzrok Ophthalmological Clinic, 80-402 Gdańsk, Poland (A.J.-M.) (A.J.-J.) (M.K.); 2 Department of Ophthalmology, University of Warmia and Mazury, 10-719 Olsztyn, Poland; 3 Institute for Research in Ophthalmology, 60-554 Poznan, Poland

**Keywords:** central serous chorioretinopathy, subthreshold micropulse laser, subretinal fluid, photodynamic therapy

## Abstract

Background: It has been recommended that any invasive treatment performed in patients with central serous chorioretinopathy (CSCR) not be initiated earlier than four months after disease onset due to the potential for spontaneous remission of symptoms. The goal of this study was to examine the outcome of transfoveal subthreshold micropulse laser treatment (SMPLT) of CSCR performed at six months or less after disease onset. Materials and methods: The study included 32 cases of CSCR lasting between three weeks and six months (mean: 3.4 ± 2.3 months). All patients had transfoveal SMPLT applied and were followed for at least three months after each session of SMPLT. Two sessions of SMPLT in total were planned in case of an insufficient response to the first instance of treatment. Evaluation parameters included any change in best-corrected visual acuity (BCVA) and retinal morphology. Results: Total resolution of subretinal fluid (SRF) was noted in 26 cases (81.25%). Final BCVA improved significantly from 0.37 ± 0.22 logMAR to 0.22 ± 0.20 logMAR after treatment. Overall, early SMPLT correlated with better final BCVA (*p* = 0.0005, Spearman rank correlation). For eyes achieving a total resolution of SRF, BCVA improved from 0.33 ± 0.21 logMAR to 0.17 ± 0.14 logMAR (*p* = 0.004, Spearman rank correlation). The analysis of SMPLT nonresponders revealed a tendency for poorer baseline visual acuity. Conclusions: Patients with CSCR lasting six months or less treated with transfoveal SMPLT achieve better functional results with early application of this procedure. As baseline BCVA predicts final visual acuity, earlier treatment, permitted by the safety of SMPLT, may improve final visual outcomes.

## 1. Introduction

Central serous chorioretinopathy (CSCR) is a well-known and quite common clinical entity; however, its pathogenesis remains unclear [1]. In many acute cases, symptoms recede spontaneously without serious visual impairment. Still, some retinal changes can be observed after acute CSCR (e.g., retinal pigment abnormalities), and patients may complain about a decline or change in the quality of their vision, especially in the context of persistent metamorphopsia and differences in visual acuity between the eyes. The chronic form of CSCR in particular may be a serious therapeutic problem, often leading to significant visual impairment and a reduced quality of life [2]. It is for this reason why different therapeutic methods have been introduced in an attempt to solve the problem. Traditionally, it has been recommended to wait for four months for a spontaneous resolution of symptoms before the application of any invasive medical treatment [3]. This guideline was due to both the tendency of many acute cases to resolve spontaneously and the risks of conventional macular photocoagulation (LPC) and full-fluence photodynamic therapy (PDT). LPC is used in selected cases where the leakage point is found to be located at a safe distance from the center of the fovea. Conversely, PDT may be used closer to the center of the fovea, with its safety improved by reducing the fluence of the photo-activating laser. The application of PDT is usually delayed until six months from the beginning of symptoms in expectance of spontaneous remission, as it involves the invasive procedure of the intravenous injection of verteporfin and potentially troublesome necessity to avoid sunlight after the therapy [4,5,6]. Systemic treatment, such as oral mineralocorticoid receptor inhibitors (e.g., eplenerone), may be effective as well, but clinical data on such medications are thus far limited in number [7,8].

The advent of micropulse lasers has changed the approach to the treatment of CSCR. Subthreshold micropulse laser treatment (SMPLT) has been successfully used in chronic CSCR with morphological success achieved in the majority of cases [9,10,11,12]. This procedure is comfortable for the patient and not especially expensive. Stimulation of the retinal pigment epithelium (RPE) by SMPLT results in the production of so called ‘heat shock proteins’(HSP), which are mainly cytokines and chaperones. HSPs have a multitude of effects including anti-inflammatory and antiangiogenic properties [13,14,15]. By normalizing RPE function, SMPLT improves the transretinal pump to eliminate subretinal fluid (SRF). Energy is delivered to the tissue in a series of very short impulses, between which there are intervals that enable the tissue to cool down, preventing heat accumulation to a level that is lethal to the RPE. Thus, SMPLT has been proven to be a safe procedure, without known adverse treatment effects [16,17]. Unfortunately, in chronic CSCR, the morphological success of this treatment may not be accompanied by a significant improvement in visual acuity. Instead, the gain in visual acuity is typically small, usually equivalent to one line on a Snellen chart [18,19,20,21]. By definition, such poorer visual outcomes in chronic CSCR are attributable to the longer duration of the disease, which leads to increased damage to the photoreceptors and retinal thinning.

The proven safety of SMPLT allows for the consideration of earlier treatment of patients with CSCR. The goal of our study was therefore to relate functional and morphological outcomes of transfoveal SMPLT to the timing of the initiation of SMPLT therapy. As permanent retinal damage from CSCR is more common in eyes with a duration of disease of six months or more, the current study focused on eyes with documented disease durations of less than six months to learn how earlier intervention may influence long-term outcomes.

## 2. Experimental Section

All procedures performed in this study were done in accordance with the ethical standards of the institutional research committee and the 1964 Declaration of Helsinki. Written consent for the procedure was obtained in all cases.

This case series study included 32 patients with CSCR, extracted from the group of 66 patients treated with SMPLT for CSCR in Dobry Wzrok Ophthalmological Clinic between January 2015 and January 2018. Inclusion criteria were the duration of symptoms up to 6 months, presence of active disease defined by the presence of SRF on spectral domain optical coherence tomography (SOCT), subfoveal location of SRF and lack of choroidal neovascularization (CNV).

All patients were treatment-naive. There was no medical history of systemic or topical steroid intake. Baseline examination included best-corrected visual acuity (BCVA) check, slit lamp examination and ophthalmoscopy, SOCT examination (Zeiss Cirrus 4000; Carl Zeiss AG, Oberkochen, Germany), fluorescein angiography (FA; Zeiss FF-450; Carl Zeiss AG, Oberkochen, Germany), and fundus autofluorescence (FAF; Zeiss FF-450; Carl Zeiss AG, Oberkochen, Germany). BCVA was tested by the optometrist, who was not involved in the therapeutic procedures (blinded examiner). For statistical purposes, the BCVA results were converted to logMAR values. In cases suspected to have choroidal neovascularization, OCT angiography was additionally performed (REVO Copernicus; Optopol Technology, Zawiercie, Poland), and patients with confirmed CNV were subsequently excluded from the study.

SOCT measurements included central retinal thickness (CRT) and average central retinal thickness (CRTA). Both measurement options are included in the SOCT software. CRT refers to an average retinal thickness within the central circle of 1 mm in diameter, while CRTA refers to an average retinal thickness within the central circle of 6 mm in diameter. SRF height was measured manually at its highest point using software provided by the SOCT manufacturer.

Follow-up examinations were scheduled for all participants at two months after each session of SMPLT and six months after initial inclusion in the study. The follow-up visits included undergoing BCVA examinations, SOCT scans, and fundus autofluorescence (FAF) imaging. If SRF was still present at two months, a second SMPLT session was performed within 30 days (for a total three-month range of evaluation sessions). Thus, two SMPLT sessions in total were performed before the final assessment.

SMPLT was performed with a 577 nm laser (Supra Scan 577; Quantel Medical, Cournon d’Auvergne, France). The procedure was carried out according to the SOCT map, in that the whole SRF area was covered with confluent laser foci of 160 µm in diameter. As the SRF involved the fovea in each case, the fovea was included in SMPLT. Fixed laser parameters of 250 mW, a duty cycle at 5%, and a 0.2 s duration were employed in each case. At each follow-up visit, a FAF image was taken to identify any laser-induced damage to the RPE.

### Statistical Analysis

Statistical analysis included the efficacy of SMPLT and the relationship between the results of treatment and the time point of therapy initiation. Additional evaluation included the parameters of retinal morphology and BCVA before and after treatment.

The parameters of the morphological efficacy of SMPLT were the percentage of patients with a total resolution of SRF and the changes in CRT and CRTA. Functional success was defined by the improvement in BCVA that was statistically significant.

The final values of CRT, CRTA, SRF height, and BCVA were correlated with the duration of the disease before the initiation of treatment.

Statistical analysis was completed with the use of the Statistica 10.0 software (StatSoft, Tulsa, OK, USA). The normalcy of distribution was assessed using the Shapiro–Wilk test. The significance of the results of treatment was evaluated using the Wilcoxon test. Correlations between the duration of symptoms and the results of treatment were assessed by calculations of Spearman rank coefficients.

Comparisons between groups were evaluated by the nonparametric Mann–Whitney U test. The results considered statistically significant when the calculated probability satisfied the inequality test (*p* < 0.05).

## 3. Results

Disease duration in the studied group ranged from three weeks to six months, 3.36 ± 2.26 months on average (median value 4.0, IQR = 5.0). There were 11 females and 21 males in the study group, with a mean age of 48.2 ± 11.0 years. Age distribution and diversity of the duration of the disease are presented in the Table 1.

The total resorption of SRF was achieved in 26 cases (81.25%). A second session of SMPLT was performed in eight cases—of which two cases responded well, showing a total resolution of SRF, while SRF continued to persist in the remaining six cases.

Baseline characteristics of the study group and the results of treatment are presented in Table 2.

The differences between all parameters before and after treatment were statistically significant according to the Wilcoxon test (*p* < 0.001 for every pair).

The analysis of the correlation between the duration of the disease and different variables characteristic of retinal morphology and function revealed a strong statistical link with BCVA after treatment only (Table 3). The amount of BCVA change after treatment borders on statistical significance (*p* = 0.07).

The difference in final BCVA between patients treated early and late is particularly clearly visible upon dividing the whole study group into two subgroups of patients who had undergone SMPLT within two months of the onset of symptoms and patients who were treated at between two and six months after onset of the disease, respectively. The difference between BCVA logMAR values for these subgroups is statistically significant according to the Mann–Whitney U test (*p* = 0.00003) (Table 4).

Statistical analysis of the subgroup of patients who achieved a resolution of SRF after SMPLT revealed similar results as analysis of the whole cohort of patients. Baseline characteristics of this subgroup is presented in Table 5.

The observed changes in all parameters after treatment were statistically significant (*p* < 0.001 for all pairs). The analysis of correlations between the duration of symptoms and parameters after treatment revealed a strong relation to final BCVA: a shorter duration of CSCR was associated with better final visual acuity. Results are presented in Table 6.

For this cohort, the relationship between the earlier initiation of treatment and better functional results is also clearly visible, including with the division into the two subgroups (Table 7).

The comparison of baseline characteristics between good responders and nonresponders to SMPLT, respectively, revealed no statistically significant differences, but it should be recalled that the nonresponders group was small in size, containing only six eyes. Nevertheless, the baseline BCVA values in nonresponders were lower (0.52 ± 0.21 logMAR vs. 0.33 ± 0.21 logMAR), and this difference borders on statistical significance (*p* = 0.06). With a larger population sample, this result could become statistically significant. Results of this analysis are presented in Table 8.

Treatment safety was assessed by FAF after each session of SMPLT. At any point in the study, no detectable damage to the RPE as a result of subthreshold laser application was noted in any participants.

## 4. Discussion

The optimal timing of CSCR treatment remains unclear and, due to the emergence of new treatment options, has become a topic of renewed interest [22,23]. Traditionally, it has been recommended that invasive treatments be avoided in patients with acute CSCR in consideration of the risks of treatment and generally good outcomes seen with observation only. Moreover, these recommendations have considered PDT as the only effective treatment for chronic CSCR [24,25]. Placing LPC spots close to the fovea usually resulted in scotomas or metamorphopsia and increased the risks of inadvertent foveal injury and CNV; thus, LPC application is generally delayed, reserved only for persistent cases. Nowadays, we realize that acute CSCR is also connected with some retinal tissue damage, although the exact moment of the onset of this injury is not precisely defined. It has been shown that the correlation between the duration of CSCR and the degree of visual impairment is not linear [18].

We believe that there are three main factors that support reconsideration of the standard recommendations for CSCR treatment with regard to both the timing and mode of treatment. First, micropulse laser technology, which provides retinal treatment, is substantially safer than classical laser photocoagulation, having no known adverse treatment effects in any clinical application. Second, the functional results of CSCR treatment are strongly connected with the duration of the disease. Patients treated earlier recover good visual acuity and experience restored retinal morphology. In contrast, none of the treatments of chronic CSCR have provided satisfactory functional results. Scholz et al., in a meta-analysis of results of the treatment of chronic CSCR by SMPLT from 12 studies, found an average improvement in BCVA of 6.34 ETDRS letters (range: −15 to +20) [26]. Poor functional improvement is also attributed to standard PDT therapy for chronic CSCR. Mohabhati et al. reported an average improvement of six ETDRS letters in the ‘severe’ chronic CSCR group and four ETDRS letters in the ‘standard’ chronic CSCR group [27]. The same applies to the large PLACE trial (Half-Dose Photodynamic Therapy versus High-Density Subthreshold Micropulse Laser Treatment), where average improvements of BCVA after half-dose PDT were reported at 4.6 ETDRS letters in chronic CSCR [6]. Oral eplerenone therapy in chronic CSCR, when effective at all, provided a similar small rate of BCVA improvement in most of the existing studies [15,28,29].

Third, we know that retinal architecture can be normalized after SMPLT in the majority of patients including with significant reductions in retinal thickness and the resolution of SRF [9,10,11,12,18,20,21]. If retinal architecture can be improved, then, theoretically, functional improvement may follow. Permanent retinal damage, more likely to occur in chronic cases, may limit the potential for both treatment response and visual recovery. Therefore, although most cases of acute CSCR resolve spontaneously, effective early treatment that is able to be provided safely may improve long-term outcomes by reducing the risk of chronic CSCR and thus permanent visual impairment.

Among the current therapeutic options, SMPLT is the simplest, safest, and least invasive choice. PDT requires the intravenous injection of verteporfin, and the patient is advised to avoid sunlight exposure soon after the therapy due to possible skin photosensitivity reactions. Eplerenone therapy has systemic side effects including gynecomastia in males and requires the systematic control of potassium plasma levels during such treatment. In comparison, at this time, SMPLT has no known adverse treatment effects or limitations, as illustrated by the safety of direct foveal treatment in this and prior studies [30,31,32].

Of note, we are not the first group to report on the efficacy of SMPLT in early CSCR. Luttrull et al. analyzed the effects of SMPLT in a small group of 11 cases with durations of between one and seven months, finding a total resolution of SRF occurred in all cases regardless of disease duration [33]. Scholz et al., in their two studies on chronic CSCR, included patients with disease durations of as short as six weeks or longer among larger populations of 38 and 42 eyes [19,20]. In the current study, we wished to further examine the influence of early treatment on functional and/or morphological outcomes. Our results demonstrate that even patients who respond well to treatment anatomically and who achieve a total resolution of SRF may end up with residual impairments of their BCVA. In the current study, we identified a strong correlation between final BCVA and the duration of the disease before the initiation of treatment, even in eyes enjoying a total resolution of SRF. Patients who had SMPLT performed within two months of the onset of symptoms had significantly better final visual acuity than did those in whom SMPLT was performed later. As our study shows, irreversible damage to photoceptors can occur earlier than at four months of disease duration, which is the traditionally accepted time for the initiation of treatment. The traditional definition of chronic CSCR refers only to disease duration and does not account for the nature of RPE decompensation (i.e., focal, diffuse, or indistinct) or disease damage to the retina and/or RPE at presentation. These are the main clinical factors that influence the anatomic and functional responses to treatment [34,35,36,37]. Thus, the traditional acute versus chronic dichotomy applied to CSCR fails to accurately capture disease complexity in important ways. In our study, nonresponders to SMPLT did not have a significantly longer duration of the disease in comparison with good responders but did tend to have worse visual acuity at presentation.

In light of these findings, it appears that, even in cases of self-limiting CSCR, potentially visually limiting retinal damage begins with disease onset and worsens as the disease persists. This observation supports the push to minimize disease duration, provided it can be done simply and safely. SMPT permits such early intervention [13,14,16].

The most distinct advantage of treating acute cases of CSCR was presented in the study by Arora et al. [22]. The authors treated acute cases of CSCR with durations of shorter than two months and compared the functional results of treatment with those in observation-only cases. Treated cases had better BCVA values and contrast sensitivity than did the observed cases throughout six months of follow-up. Here, the mean BCVA at six months was 0.03 logMAR in the laser group and 0.14 logMAR in the observation group. This difference was highly significant (*p* = 0.008). Separately, the mean contrast sensitivity at six months was 1.65 logMAR in the laser group and 1.45 logMAR in the observation group. This difference favored SMPLT as well (*p* < 0.001). Furthermore, these improvements resulting from prompt SMPLT in CSRS were achieved without any adverse treatment effects, consistent with prior studies [11,38,39,40].

Our findings thus suggest that early treatment of CSCR with SMPLT is effective, with the potential for improving long-term functional outcomes. Confirmation by larger prospective studies appears to be warranted.

### Limitations of the Study

We realize that our study is based on a relatively small sample, but, nevertheless, the study population size was sufficient enough to show the difference in functional outcomes of the treatment. Further studies involving larger groups of patients are, however, needed, especially involving the evaluation of retinal function beyond BCVA (microperimetry or multi-focal electroretinography). We also realize that we present a case series study, not the randomized controlled trial. In the future, it is necessary to confront these results with the outcome of prospective studies. Longer follow up could also provide information about the recurrence rate after SMPLT.

## 5. Conclusions

Our study suggests that patients with acute CSCR may benefit from early treatment with SMPLT. The application of this therapy within the first weeks of the disease promoted better functional improvement in comparison to later treatment.

## Figures and Tables

**Table 1:** Characteristics of the study group.

**Parameter**	**N**	**Mean**	**Median**	**Minimum**	**Maximum**	**SD**
Age	32	48.19	48.00	30.00	71.00	11.02
Duration of symptoms (months)	32	3.36	4.00	0.60	6.00	2.26
**Parameter**	**N**	**Median**	**Lower quartile**	**Upper Quartile**	**Interquartile range**	
Duration of symptoms (months)	32	4.00	1.00	6.00	5.00	

**Table 2:** Study group baseline characteristics and results of subthreshold micropulse laser treatment (SMPLT).

Parameter	N	Mean	Median	Minimum	Maximum	SD
(I) CRT (µm)	32	372.69	367.50	248.00	658.00	89.59
(II) CRT (µm)	32	262.47	248.00	167.00	471.00	57.74
(I) SRF height (µm)	32	177.66	146.00	70.00	417.00	85.28
(II) SRF height (µm)	32	23.06	0.00	0.00	304.00	61.50
(I) CRTA (µm)	32	311.31	305.00	254.00	413.00	33.79
(II) CRTA (µm)	32	290.97	294.00	246.00	332.00	16.22
(I) BCVA logMAR	32	0.37	0.40	0.10	1.00	0.22
(II) BCVA logMAR	32	0.22	0.20	0.00	0.80	0.20
No EXP	32	318.41	275.00	120.00	648.00	154.24

N—number of eyes, SD—standard deviation, CRT—central retinal thickness, CRTA—average central retinal thickness, BCVA—best-corrected visual acuity, SRF—subretinal fluid, EXP—number of laser impacts per session, I—results before treatment, and II—results after treatment.

**Table 3:** Spearman correlation rank test for the relationship between the duration of CSCR and the parameters of retinal morphology and function after treatment.

Parameter	N	R Spearman	T(N-2)	*p*
(II) CRT (µm)	32	0.0987	0.5430	0.5911
(II) SRF (µm)	32	0.2626	1.4909	0.1464
(II) CRTA (µm)	32	0.0100	0.0550	0.9565
(II) BCVA logMAR	*32*	*0.5802*	*3.9014*	*0.0005*
BCVA logMAR (I–II)	32	−0.3243	−1.8775	0.0702
CRT (I–II)	32	−0.0392	−0.2149	0.8313
CRTA (I–II)	32	−0.0818	−0.4496	0.6562

I–II—difference between parameter before and after treatment.

**Table 4:** LogMAR values of final BCVA for the subgroups of patients according to the time point of initiation of treatment.

Timing of Treatment	N	Mean	Median	Minimum	Maximum	SD
2 months and earlier	15	0.09	0.10	0.00	0.30	0.09
2–6 months	17	0.34	0.20	0.20	0.80	0.20

**Table 5:** Baseline characteristics and results of SMPLT in the subgroup of patients with a total resolution of subretinal fluid (SRF).

Parameter	N	Mean	Median	Minimum	Maximum	SD
(I) CRT (µm)	26	375.73	371.50	248.00	658.00	98.06
(II) CRT (µm)	26	239.69	240.00	167.00	276.00	24.38
(I) SRF (µm)	26	180.73	148.50	70.00	417.00	88.47
(I) CRTA (µm)	26	313.00	305.50	254.00	413.00	36.63
(II) CRTA (µm)	26	288.42	291.00	246.00	317.00	15.67
(I) BCVA logMAR	26	0.33	0.30	0.10	1.00	0.21
(II) BCVA logMAR	26	0.17	0.20	0.00	0.60	0.14
No EXP	26	320.46	310.00	120.00	575.00	145.17

**Table 6:** Spearman correlation rank test for the relationship between the duration of CSCR and the parameters of retinal morphology and function after treatment in patients with a total resolution of SRF.

Parameter	N	R Spearman	T(N-2)	*p*
(II) CRT (µm)	26	−0.1382	−0.6834	0.5009
(II) CRTA (µm)	26	−0.1316	−0.6506	0.5215
(II) BCVA logMAR	26	0.5444	3.1793	0.0040
BCVA logMAR (I−II)	26	−0.3629	−1.9080	0.0684
CRT (I−II)	26	0.1414	0.7000	0.4907
CRTA (I−II)	26	0.0404	0.1981	0.8447

**Table 7:** LogMAR values of final BCVA in patients with a total resolution of SRF for the subgroups of patients according to the time point of the initiation of treatment.

Timing of Treatment	N	Mean	Median	Minimum	Maximum	SD
Two months and earlier	15	0.09	0.10	0.00	0.30	0.09
Two to six months	11	0.26	0.20	0.20	0.60	0.13

The differences between BCVA values in these subgroups were statistically significant (*p* = 0.0004).

**Table 8:** Comparison between the baseline parameters of good responders and nonresponders to SMPLT according to the Mann–Whitney U test.

Parameter	N	N	*p*
Duration (months)	26	6	0.16
CRT (µm)	26	6	0.80
SRF (µm)	26	6	0.69
CRTA (µm)	26	6	0.83
*BCVA logMAR*	*26*	*6*	*0.06*
No EXP	26	6	0.76
